# Diagnosis of Prosthetic Joint Infection of Hips and Knees—One Size Does Not Fit All

**DOI:** 10.1093/ofid/ofaf195

**Published:** 2025-03-28

**Authors:** Anne Spichler-Moffarah, Lauren Daddi, Duc Nguyen, Ilda Molloy, Marjorie Golden

**Affiliations:** Division of Infectious Diseases, Department of Internal Medicine, Yale School of Medicine, New Haven, Connecticut, USA; Yale School of Medicine, New Haven, Connecticut, USA; Division of Infectious Diseases, Department of Internal Medicine, Yale School of Medicine, New Haven, Connecticut, USA; Veteran Affairs Connecticut Healthcare System, West Haven, Connecticut, USA; Department of Orthopedics & Rehabilitation, Division of Adult Reconstruction, Yale University School of Medicine, New Haven, Connecticut, USA; Division of Infectious Diseases, Department of Internal Medicine, Yale School of Medicine, New Haven, Connecticut, USA

**Keywords:** classification, criteria, prosthetic joint infection, scoring, diagnostics

## Abstract

There is no consensus on diagnostic criteria for hip and knee periprosthetic joint infections (PJIs). This study evaluated Infectious Diseases Society of America (IDSA), International Consensus Meeting 2018 (ICM2018), and European Bone and Joint Society (EBJIS) criteria, finding IDSA most accurate for early PJI and knee infections, while ICM2018 and EBJIS were superior for delayed and late cases. Diagnostic approaches should consider infection timing and context.

Periprosthetic joint infection (PJI) is a well-documented complication of joint arthroplasty, with incidence rates of 0.8%–2.18% for the knees and 0.3%–2.18% for the hips [1–4]. Diagnosis of PJI depends on various factors, including host characteristics, the causative microorganisms, and the timing relative to implantation [3, 5]. Accurate and consistent diagnostic criteria are critical for ensuring appropriate management of PJI [5–7].

Scoring systems have been created by the Infectious Diseases Society of America (IDSA), International Consensus Meeting 2018 (ICM2018), and European Bone and Joint Society (EBJIS) that combine clinical findings, laboratory parameters (from blood and synovial fluid), culture, and histological analyses [8–10]. C-reactive protein (CRP), erythrocyte sedimentation rate (ESR), alpha-defensin, and culture of hardware sonicate all help establish the diagnosis [1, 6, 11, 12].

Data to inform which criteria perform best and which are therefore most useful in clinical practice are limited [11–13]. Current scoring systems fail to account for the time from arthroplasty to diagnosis and duration of symptoms. This study evaluates the performance of the 3 scoring systems for PJI in hips and knees, focusing on acute, delayed, and late presentations.

## METHODS

### Study Design and Patient Selection

This retrospective study included patients admitted to Yale-New Haven Hospital (YNNH) between September 2017 and December 2020 with first PJI of the hip or knee. Inclusion criteria were age ≥18 years and surgical management of PJI.

Exclusions included patients managed only as outpatients, transfers from external institutions, and prior PJI. Subjects were identified by the Yale Center for Clinical Investigation Joint Data Analytics Team using specific International Statistical Classification of Diseases, 10th Revision (ICD-10), codes for PJI of the hip or knee.

### Data Collection

Two infectious diseases physicians, in consultation with orthopedic surgeons, conducted comprehensive manual chart reviews. Collected variables included clinical findings (sinus tract, intra-articular purulence, fever), microbiology (intraoperative cultures including sonication), synovial aspiration data (nucleated counts, differential, alpha-defensin), and inflammatory markers (ESR, CRP). Demographic data, time from index arthroplasty to PJI diagnosis, and time from diagnosis to surgery were recorded.

### Definitions

The IDSA, ICM2018, and EBJIS scoring systems are well described [8–10]. Please see Supplementary Table 1 for reference. The period between arthroplasty to infection is classified as early (<90 days), delayed (90 days–2 years), and late (>2 years) [5].

### Ethics

The study protocol was approved by Yale University's Institutional Review Board.

## Statistical Analysis

Analysis of variance (ANOVA) compared numerical variables, while chi-square tested categorical variables. Tukey's honestly significant difference test was used to compare numerical variables after analysis of variance. Receiver operating characteristic (ROC) curves with area under the curve (AUC) were generated via logistic regression to evaluate IDSA, ICM2018, and EBJIS criteria for early, delayed, and late PJI diagnosis, controlling for age, gender, and body mass index. Data were analyzed using SAS 9.4 (SAS Institute, Cary, NC, USA).

## RESULTS

A total of 140 patients were diagnosed with hip or knee PJI based on diagnostic criteria from at least 1 of the 3 scoring systems. Subjects were categorized as early (n = 52), delayed (n = 24), and late (n = 64) PJI. Early and delayed cases were more common in females. Most patients were White (77%) and non-Hispanic (95%), with infections affecting knees (56%) more than hips (45%). Table 1 summarizes demographics, clinical characteristics, and criteria distribution by presentation timing.

**Table 1.** Demographic, Clinical Characteristics, and PJI Criteria by PJI Classification

Characteristics n, (%)	All Patients	0–3 Months	3–24 Months	>24 Months	*P* Value
		(Early PJI)	(Delayed PJI)	(Late PJI)	
	n = 140	n = 52 (37)	n = 24 (17)	n = 64 (46)	…
Age, mean ± SD, y	69.5 ± 12.8	65.5 ± 13.4	66.1 ± 11.8	74.1 ± 11.0	.0004
Gender, No. (%)	…	…	…	…	.4
Male	61 (43.6)	19 (36.5)	12 (50)	34 (53.1)	
Female	79 (56.4)	33 (63.5)	12 (50)	30 (47.9)	
Race, No. (%)	…	…	…	…	.03
White or Caucasian	108 (77.1)	39 (75)	18 (75)	51 (79.7)	
Black or African American	24 (17.1)	9 (17.3)	2 (8.3)	13 (20.3)	
Other/unknown	8 (5.7)	4 (7.7)	4 (16.7)	0	
Ethnicity, No. (%)	…	…	…	…	.04
Non-Hispanic	133 (95)	50 (96.2)	20 (83.3)	63 (98.4)	
Hispanic or Latino	7 (5)	2 (3.8)	4 (16.7)	1 (1.6)	
BMI, median (IQR), kg/m^2^	30 (26–36)	26 (24–31)	32 (26–36)	29 (25–33)	.64
PJI location, No. (%)	…	…	…	…	.0003
Hips	61 (43.6)	34 (65.4)	6 (25)	21 (32.8)	
Knees	79 (56.4)	18 (34.6)	18 (75)	43 (67.2)	
Time from symptoms to diagnose, median (IQR), d	7 (3–18)	4.5 (3–9.5)	7 (4–28)	9 (3.5–30)	.03
Time from symptoms to surgery, median (IQR), d	9 (4–21)	5.5 (4–13)	10 (6–43.5)	12 (5–42.5)	.04
Clinical, No. (%)	…	…	…	…	…
Fever	56 (40)	24 (46.2)	8 (33.3)	24 (37.5)	.5
Purulence in the joint	57 (40.7)	16 (30.8)	11 (45.8)	30 (46.9)	.2
Sinus tract	20 (14.3)	10 (19.2)	4 (16.7)	6 (9.4)	.3
Laboratory, No. (%)	46 645 (21 025–90 850)	56 400 (5000–76 000)	49 500 (27 205–95 500)	45 622 (22 050–105 900)	.9
Synovial nucleated, median (IQR)	91 (84–95)	93.5 (80–96.5)	90.5 (83–94)	90 (85–95)	…
Neutrophils, median (IQR), %	118 (35–204)	118 (45–222)	61 (16–165)	153 (35–204)	…
CRP, mg/L	74 (43–100)	70 (34–93)	79 (43–107)	75 (49–101)	.3
ESR, mm/h	…	…	…	…	.4
PJI criteria, No. (%)	…	…	…	…	
IDSA	…	…	…	…	
Yes	120 (85.7)	48 (92.3)	20 (83.3)	52 (81.3)	
Yes, clinical	19 (13.6)	4 (7.7)	4 (16.7)	11 (17.2)	
No	1 (0.7)	0	0	1 (1.6)	
ICM	…	…	…	…	
Infected	124 (88.6)	43 (82.7)	22 (91.7)	59 (92.2)	
Possible	15 (10.7)	9 (17.3)	2 (8.3)	4 (6.3)	
Not infected	1 (0.7)	0	0	1 (1.6)	
EBJIS	…	…	…	…	
Confirmed	128 (91.4)	45 (86.5)	22 (91.7)	61 (95.3)	
Likely	10 (7.1)	6 (11.5)	1 (4.2)	3 (4.7)	
Unlikely	2 (1.4)	1 (1.9)	1 (4.2)	0	
All 3 criteria (confirmed or likely/possible)	136 (97.1)	51 (98.1)	23 (95.8)	62 (96.9)	
IDSA + ICM	138 (98.6)	52 (100)	24 (100)	62 (96.9)	
IDSA + EBJIS	137 (97.9)	51 (98.1)	23 (95.8)	63 (98.4)	
ICM + EBJIS	137 (97.9)	51 (98.1)	23 (95.8)	63 (98.4)	

Table values are mean (SD) for continuous variables and No. (column %) for categorical variables; column percentages may not sum to 100% due to rounding. Analysis of variance was used to compare numerical variables (age, BMI, and laboratory data). The chi-square statistic was used to compare categorical variables. Tukey's honestly significant difference test was used to compare numerical variables after analysis of variance: Age in the late PJI group was significantly higher than age in the early and delayed PJI group, and time from symptoms to surgery and time from symptoms to diagnosis were longer in the late PJI group vs the early PJI group.

Abbreviations: BMI, body mass index; EBJIS, European Bone and Joint Infection Society criteria for PJI; ESR, erythrocyte sedimentation rate; ICM, International Consensus Meeting criteria for PJI; IDSA, Infectious Diseases Society of America criteria for PJI; IQR, interquartile range; PJI, prosthetic joint infection.

The median time from symptom onset to PJI diagnosis was 7 days (4.5 days for early and 9 days for late PJI). Time from diagnosis to surgery was 5.5 days for early, 10 days for delayed, and 12 days for late PJI (Table 1). Clinical characteristics (fever, purulence in joint, presence of sinus tract) did not differ across early, delayed, and late infections.

Intraoperative cultures were positive in 80% of cases, with higher likelihood of culture identification in early PJI compared with delayed or late PJI. Sonicate cultures, performed in 82% of patients, had some growth (70% cases), including some cases with <10 CFU/mL, which may not be of clinical significance. The results of preoperative aspiration culture positivity were similar across all criteria. Bacteremia was more frequent in late PJI.

The most common pathogens in early PJI were methicillin-sensitive *Staphylococcus aureus* (MSSA), coagulase-negative staphylococci (CoNS), *Cutibacterium acnes*, and *Enterococcus* species. Delayed PJI commonly involved MSSA, CONS, and *Streptococcus* species, while late PJI was often caused by CONS, MSSA, *Streptococcus* species, and *C. acnes*.

**Figure 1.**
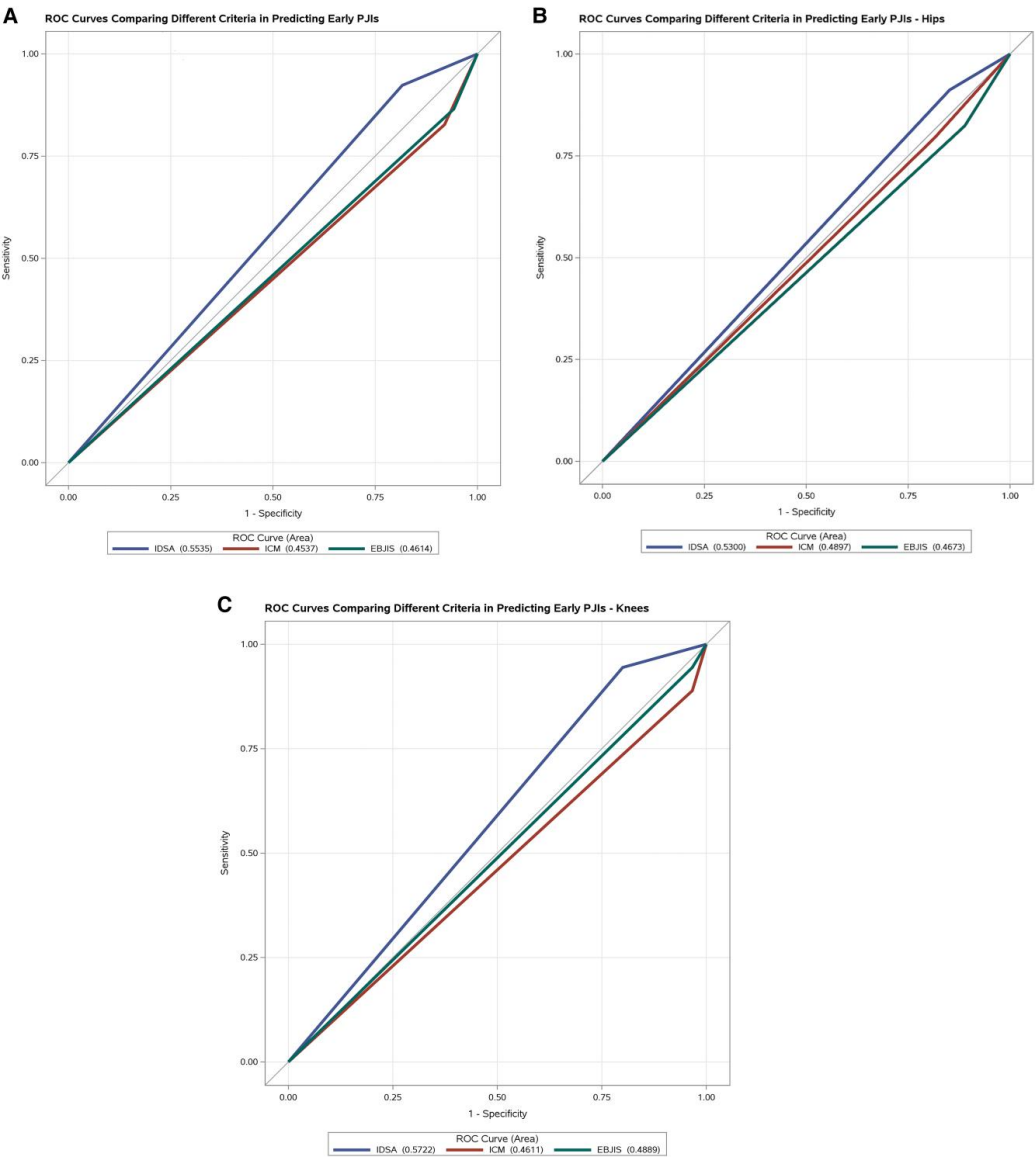
ROC curves comparing how different criteria (IDSA, ICM, EBJIS) diagnose early (<90 d) PJI for all infections (*A*) and separately for hip (*B*) and knee (*C*) infections. AUC was calculated for each criterion. ROC and AUC were calculated using logistic regression. Abbreviations: AUC, area under the curve; EBJIS, European Bone and Joint Society; ICM, International Consensus Meeting; IDSA, Infectious Diseases Society of America; PJI, periprosthetic joint infection; ROC, receiver operating characteristics.

Overall, 85.7% of patients met IDSA criteria for confirmed PJI, 88.6% met ICM2018 criteria, and 91.4% met EBJIS criteria. The IDSA criteria were most accurate for early PJI diagnosis, while the ICM2018 and EBJIS criteria performed better for delayed and late PJI. The IDSA criteria showed higher accuracy for knee PJI diagnosis. Figure 1A–C shows ROC curves for predicting PJI based on site and timing of infection for early PJI.

## DISCUSSION

Our study highlights differences in the performance of diagnostic criteria for PJI based on timing of infection following arthroplasty and affected site. IDSA criteria were more accurate for early PJI diagnosis and knee PJI. ICM2018 and EBJIS performed better for delayed or late PJI cases, with little influence from the time of symptoms to diagnosis. These findings support tailoring diagnostic approaches to the timing and context of infection.

Previous studies evaluating diagnostic criteria for PJI focus on sensitivity and specificity but do not mention time from arthroplasty to PJI diagnosis [11–13]. IDSA criteria rely on presence of a sinus tract communicating with the prosthesis, acute inflammation on histological examination, purulence, positive cultures, and growth of a virulent microorganism; they do not include inflammatory markers, synovial characteristics, imaging, or alpha-defensin [9]. Given that early PJI is often characterized by an acute inflammatory response, IDSA criteria may be more prone to detect infection with rapid onset, as well as early PJI. In contrast, delayed or late PJIs often require additional diagnostic tools beyond the IDSA framework due to their potential indolent presentation.

In our study, patients with early and delayed PJI had a higher prevalence of MSSA than late PJIs, raising the possibility that patients with early PJI had more virulent organisms, which are more likely to yield positive culture results. IDSA criteria also allow for clinical judgment to make the determination of PJI. When we looked at patients diagnosed solely based on clinical judgment (1 of the IDSA criteria), most had delayed (16.7%) and late (17.2%) PJI, again suggesting that in early PJI clear signs of infection are often present. For late and delayed PJI, EBJIS and ICM2018 [8, 10] performed better, possibly due to use of a larger number of parameters in all criteria categories such as “confirmed” and “infected” and “likely infected” and “possibly infected,” respectively.

We acknowledged the inconsistencies in defining early, delayed, and late PJI, with some classifications using 30 days, others 90 days, making standardization challenging [14]. We chose the Zimmerli classification [5] for its clinical utility, as it accounts for both postoperative and hematogenous infections.

This is particularly relevant given that infections occurring in the intermediate timeframe (eg, 6–8 weeks) may exhibit characteristics of both early and delayed infections. The variability in classification impacts the performance of diagnostic criteria, as inflammatory markers fluctuate across infection stages. Given the lack of stratified studies on PJI diagnostics by timing, clinicians must often rely on a combination of imperfect parameters and clinical judgment. Ultimately, while IDSA and EBJIS provide structured diagnostic approaches, the heterogeneity in classification highlights the need for more standardized, evidence-based criteria that incorporate infection timing.

A limitation of our study was its retrospective design, relying on medical record review. However, most of the variables required for diagnostic criteria were consistently documented, reflecting standardized clinical practices in our institution. Our study suggests that IDSA criteria may be most valuable for diagnosis of early PJI in the knee, but further research is needed to validate these findings.

## Supplementary Material

ofaf195_Supplementary_Data

## Supplementary Data

Supplementary materials are available at *Open Forum Infectious Diseases* online. Consisting of data provided by the authors to benefit the reader, the posted materials are not copyedited and are the sole responsibility of the authors, so questions or comments should be addressed to the corresponding author.
