# Supplementary material for: Diagnosis of Prosthetic Joint Infection of Hips and Knees—One Size Does Not Fit All
Source: Open Forum Infect Dis. 2025 Mar 28;12(4):ofaf195. doi: 10.1093/ofid/ofaf195 (PMC12006793; doi:10.1093/ofid/ofaf195)
Supplement: ofaf195_Supplementary_Data [file ofaf195_supplementary_data.docx]

Supplemental Table 1: Criteria and scoring systems for 3 models of PJI diagnosis.

| IDSA | ICM | EBJIS |
| --- | --- | --- |
| ≥1 Criteria = **PJI** | ≥1 Major Criteria = **PJI**  OR  ≥6 points Minor Criteria = **Infected**   - 1. points Minor Criteria = **Possibly Infected**   0-1 points Minor Criteria = **Not Infected**  If pre-operative score is inconclusive, add intraoperative criteria to the score. | ≥1 Confirmatory Criteria = ‘**Infection** **Confirmed’**  ≥2 Other Criteria = ‘**Infection Likely’** |
| Criteria  - Sinus tract  - Histology (signs of acute inflammation on periprosthetic tissue)  - Purulence  - Culture (2 positive intraoperative cultures, OR 1 preoperative aspiration culture and 1 intraoperative culture with the same organism)  - Culture (growth of any virulent microbe in ≥1 specimen of tissue biopsy or synovial fluid)  - Clinical judgement | Major Criteria  - Sinus tract  - Culture (2 periprosthetic cultures with phenotypically identical organisms)  Preoperative Minor Criteria  - Serum CRP >10mg/l or D-dimer >30mm/h (**2 points**)  - Serum elevated ESR (**1 point**)  - Synovial WBC >3,000 cells/µl or LE ++ (**3 points**)  - Synovial alpha-defensin positive (**3 points**)  - Synovial PMN >80% (**2 points**)  - Synovial CRP >6.9mg/l (**1 point**)  Intraoperative Minor Criteria  -Histology positive (**3 points**)  -Purulence (**3 points**)  -Culture (1 positive) (**2 points**) | Confirmatory Criteria  - Sinus tract  - Synovial Leukocytes >3000 cells/µl  - Synovial PMN >80%  - Synovial alpha-defensin positive  - Culture (≥2 positive intraoperative cultures with same microorganism)  - Sonication (>50 CFU/ml any organism)  - Histology (≥5 neutrophils in 5+ HPF)  - Histology (presence of visible microorganisms)  Other Criteria  -Clinical features (Signs of loosening within 5 yrs implantation, previous wound healing problems, recent fever or bacteremia, or purulence)  -Serum CRP >10mg/l  -Synovial leukocytes >1500 cell/µl  -Synovial PMN >65%  -Culture (1 positive aspiration fluid culture)  -Culture (1 positive intraoperative culture)  -Sonication (>1 CFU/ml any organism)  -Histology (≥5 neutrophils in single HPF)  -WBC scintigraphy positive |
